# Supplementary material for: Association between whole grain food intake in Canada and nutrient intake, food group intake and diet quality: Findings from the 2015 Canadian Community Health Survey
Source: PLoS One. 2021 Jul 1;16(7):e0253052. doi: 10.1371/journal.pone.0253052 (PMC8248641; doi:10.1371/journal.pone.0253052)
Supplement: S1 Table — Data are from the Canadian Community Health Survey (CCHS) 2015. (PDF) [file pone.0253052.s001.pdf]

S1 Table. Individual Food Codes That Included the Term "Whole Wheat," "Whole Grain," or "Wholemeal" That Were Not Assigned to a Canada Food Guide (CFG) Food Group.

Individual Food Codes that included the term "whole wheat" that were not assigned to a Canada Food Guide (CFG) food group[a]	Number of CCHS 2015 participants that reported consuming this food on their day 1 24hr dietary recall
Spaghetti, cooked, whole wheat, fat added in cooking	4
Spaghetti, cooked, whole wheat, NS as to fat added in cooking	1
Macaroni, whole wheat, cooked, fat added in cooking	8
Macaroni, whole wheat, cooked, NS as to fat added in cooking	0
Spaghetti with tomato sauce and meatballs or spaghetti with meat sauce and/or meatballs, whole wheat noodles	22
Spaghetti with tomato sauce, meatless, whole wheat noodles	10
Lasagna with meat, whole wheat noodles	0
Lasagna, meatless, whole wheat noodles	0

Data are from the Canadian Community Health Survey (CCHS) 2015.

[a]We also searched for the terms "whole grain" and "wholemeal" but found no foods that were not already assigned to a Canada Food Group Food Group that contained these terms in their description.
