# Supplementary material for: Association between whole grain food intake in Canada and nutrient intake, food group intake and diet quality: Findings from the 2015 Canadian Community Health Survey
Source: PLoS One. 2021 Jul 1;16(7):e0253052. doi: 10.1371/journal.pone.0253052 (PMC8248641; doi:10.1371/journal.pone.0253052)
Supplement: S2 Table — (PDF) [file pone.0253052.s002.pdf]

S2 Table. Food Groups Used in the Current Study Based on Canada Food Guide Food Groups and Tiers.

Food Group Categories	CCHS CFG Food Groups
Whole Grains Tier 1-3	- Grains – whole tier 1-3
Whole Grains Tier 4	- Grains – whole tier 4
Fruit Tier 1-3	- Fruit other than juice tiers 1-3<br>- Fruit juice tiers 2 & 3
Fruit Tier 4	- Fruit other than juice tier 4
Vegetables tiers 1-3	- Vegetables – dark green tiers 1 -3<br>- Vegetables – deep yellow/orange tiers 1-3<br>- Vegetables – potatoes tiers 1-3<br>- Vegetables – other tiers 1-3<br>- Vegetable juice cocktail tiers 2 & 3
Vegetables tiers 4	- Vegetables – potatoes tier 4<br>- Vegetables – other tier 4
Refined grains tiers 1-3	- Grain products – non whole, enriched tiers 1-3<br>- Grain products, non whole nonenriched tiers 1-3
Refined grains tier 4	- Grain products – non whole, enriched tier 4<br>- Grain products – non whole non enriched tier 4
Dairy products and alternatives tiers 1-3	- Fluid milk and fortified soy-based products tiers 1-3<br>- Other milk alternatives – cheese/yogurt tiers 1-3
Dairy products and alternatives tier 4	- Other milk alternatives – cheese/yogurt tier 4
Meat and poultry tiers 1-3	- Meat – beef/game/organ tiers 1-3<br>- Meat other pork/veal/lamb tiers 1-3<br>- Poultry tiers 1-3
Meat and poultry tier 4	- Meat – beef/game/organ tier 4<br>- Meat other pork/veal/lamb tier 4<br>- Poultry tier 4
Processed meats tiers 1-3	- Processed meats tiers 1-3
Processed meat tier 4	- Processed meat tier 4
Fish and Shellfish tiers 1-3	- Fish tiers 1-3

**Food Group Categories**	**CCHS CFG Food Groups**
	- Shellfish tiers 1-3
Fish and Shellfish tier 4	- Fish tier 4<br>- Shellfish tier 4
Legumes, nuts and seeds tiers 1-3	- Legumes tiers 1-3<br>- Nuts and seeds tiers 1-3
Legumes, nuts and seeds tier 4	- Legumes tier 4
Eggs tiers 1-3	- Eggs tiers 1-3
Eggs tier 4	- Eggs tier 4
No CFG –discretionary foods	- Saturated and/or trans fat<br>- Beverages high in calories<br>- Alcoholic beverages<br>- High fat/high sugar foods
NoCFG – other foods and recipes	- Beverages low in calories<br>- Meal replacements<br>- Supplements e.g. energy bars<br>- Unsaturated fats and oils<br>- Water<br>- Recipes
NoCFG – foods not classified	- Uncategorized ingredients<br>- Not stated
