# Supplementary material for: Association between whole grain food intake in Canada and nutrient intake, food group intake and diet quality: Findings from the 2015 Canadian Community Health Survey
Source: PLoS One. 2021 Jul 1;16(7):e0253052. doi: 10.1371/journal.pone.0253052 (PMC8248641; doi:10.1371/journal.pone.0253052)
Supplement: S3 Table — (PDF) [file pone.0253052.s003.pdf]

S3 Table. Health Canada Daily Values for Nutrients Included in the Calculation of the Nutrient Rich Food Index 9.3.

**Nutrients included in the Nutrient Rich Food Index (NRF) 9.3**	**Daily Recommendation Based on Health Canada Daily Values used for Nutrition Labelling[a]**
Protein	50 g
Fiber	28 g
Calcium	1300 mg
Vitamin A	900 µg RAE
Vitamin C	90 mg
Vitamin D	20 µg
Iron	18 mg
Potassium	4700 mg
Magnesium	420 mg
Total sugar	100 g
Saturated fat	20 g
Sodium	2300 mg

[a] https://www.canada.ca/en/health-canada/services/technical-documents-labelling-requirements/table-daily-values/nutrition-labelling.html
