# Supplementary material for: Association between whole grain food intake in Canada and nutrient intake, food group intake and diet quality: Findings from the 2015 Canadian Community Health Survey
Source: PLoS One. 2021 Jul 1;16(7):e0253052. doi: 10.1371/journal.pone.0253052 (PMC8248641; doi:10.1371/journal.pone.0253052)
Supplement: S4 Table — Data are based on the Canadian Community Health Survey (CCHS) 2015 and are presented as mean ± standard error. Results are unadjusted. (PDF) [file pone.0253052.s004.pdf]

S4 Table. Unadjusted Daily Nutrient Intakes for Canadian Children and Adults Stratified by Whole Grain Food Intake.

	**No Whole Grain Food Intake (No-WG)**[a]		**Low Whole Grain Food Intake (Low-WG)**[a]		**Mid-Whole Grain Food Intake (Mid-WG)**[a]		**High-Whole Grain Food Intake (High-WG)**[a]		**p value for linear trend**[b]	
	Children n=3,305	Adults n=7,578	Children n=1,077	Adults n=2,044	Children n=1,085	Adults n=2,156	Children n=1,101	Adults n=2,141	Children	Adults
Energy, kcal	1809 ± 23	1878 ± 30	1604 ± 39	1749 ± 35	1772 ± 46	1896 ± 37	2006 ± 49	1978 ± 38	0.0004	0.2
Carbohydrates, g	239.0 ± 3.3	217.8 ± 2.4	212.9 ± 5.2	209.1 ± 3.6	241.7 ± 6.7	224.6 ± 5.7	273.7 ± 5.9	252.7 ± 6.2	<0.0001	0.0002
Carbohydrates, % of energy	52.7 ± 0.4	46.8 ± 0.6	53.1 ± 0.5	47.6 ± 1.0	54.2 ± 0.5	47.7 ± 0.7	54.8 ± 0.4	51.4 ± 0.8	<0.0001	<0.0001
Total sugar, g	106.8 ± 2.3	87.1 ± 1.3	99.4 ± 2.7	85.4 ± 2.1	108.9 ± 3.7	85.8 ± 3.3	112.6 ± 3.2	89.3 ± 2.9	0.1	0.7
Fiber, g	13.3 ± 0.2	14.7 ± 0.2	12.9 ± 0.3	17.4 ± 0.7	16.2 ± 0.4	19.2 ± 0.3	21.2 ± 0.4	24.8 ± 1.2	<0.0001	<0.0001
Total fat, g	65.5 ± 1.0	70.9 ± 1.7	56.9 ± 1.8	64.6 ± 1.8	62.0 ± 1.9	71.8 ± 1.8	68.6 ± 2.2	69.2 ± 1.7	0.6	0.6
Total fat, % of energy	31.6 ± 0.3	32.6 ± 0.4	31.0 ± 0.4	32.1 ± 0.7	30.5 ± 0.5	32.4 ± 0.5	29.3 ± 0.4	29.9 ± 0.6	<0.0001	<0.0001
Saturated fat, g	23.5 ± 0.5	23.3 ± 0.8	21.1 ± 0.7	21.0 ± 0.5	22.0 ± 0.8	23.1 ± 0.6	24.3 ± 0.8	21.9 ± 0.6	0.9	0.3
Protein, g	70.5 ± 1.2	79.2 ± 1.0	64.6 ± 2.3	75.3 ± 2.2	67.6 ± 2.3	80.5 ± 1.9	79.7 ± 2.6	83.2 ± 1.8	0.007	0.07
Protein, % of energy	15.6 ± 0.2	17.0 ± 0.2	15.9 ± 0.4	17.3 ± 0.2	15.3 ± 0.3	17.0 ± 0.2	15.8 ± 0.2	16.8 ± 0.2	1.0	0.6
Vitamin B_{12}, µg	4.0 ± 0.2	4.1 ± 0.2	3.7 ± 0.1	4.3 ± 0.5	3.6 ± 0.2	4.0 ± 0.2	4.0 ± 0.2	3.8 ± 0.4	0.5	0.6
Vitamin B_6, µg	1.4 ± 0.0	1.6 ± 0.0	1.3 ± 0.1	1.6 ± 0.0	1.4 ± 0.1	1.7 ± 0.0	1.7 ± 0.1	1.9 ± 0.1	<0.0001	0.003
Vitamin C, mg	114.5 ± 3.8	94.6 ± 2.6	108.1 ± 5.9	95.6 ± 3.4	114.8 ± 10.7	104.3 ± 4.2	116.4 ± 4.8	106.8 ± 4.4	0.8	0.02
Folate, µg	443.3 ± 9.5	454.6 ± 7.5	391.9 ± 18.4	416.3 ± 10.3	386.8 ± 13.2	421.3 ± 10.1	431.4 ± 14.4	434.0 ± 14.1	0.06	0.01
Folic acid, µg	140.6 ± 3.8	124.6 ± 2.4	126.0 ± 8.4	103.8 ± 3.4	111.6 ± 4.9	96.4 ± 4.2	120.8 ± 6.3	86.4 ± 3.7	0.0001	<0.0001

Vitamin D, µg	5.3 ± 0.2	4.5 ± 0.2	6.0 ± 0.3	4.8 ± 0.3	5.6 ± 0.2	5.1 ± 0.2	6.1 ± 0.3	5.1 ± 0.3	0.02	0.02
Niacin, mg	33.5 ± 0.6	39.3 ± 0.5	29.6 ± 1.2	37.0 ± 1.1	31.3 ± 1.2	39.6 ± 1.3	37.8 ± 1.4	40.6 ± 1.1	0.02	0.4
Vitamin A, µg RAE	600.9 ± 21.6	620.0 ± 35.7	613.7 ± 29.9	697.0 ± 48.4	592.5 ± 21.1	694.4 ± 24.8	627.3 ± 37.2	690.1 ± 26.3	0.6	0.2
Riboflavin, mg	1.8 ± 0.0	1.9 ± 0.0	1.8 ± 0.0	1.9 ± 0.1	1.8 ± 0.1	1.9 ± 0.0	1.9 ± 0.1	2.0 ± 0.0	0.6	0.6
Thiamin, mg	1.6 ± 0.0	1.5 ± 0.0	1.4 ± 0.0	1.5 ± 0.0	1.5 ± 0.1	1.6 ± 0.0	1.9 ± 0.1	1.8 ± 0.0	0.009	<0.0001
Sodium, mg	2561 ± 43	2760 ± 51	2216 ± 54	2509 ± 55	2408 ± 73	2703 ± 88	2760 ± 68	2866 ± 106	0.2	0.5
Potassium, mg	2332 ± 32	2593 ± 28	2212 ± 52	2662 ± 49	2396 ± 81	2784 ± 45	2670 ± 66	2960 ± 58	<0.0001	<0.0001
Zinc, mg	9.1 ± 0.2	10.2 ± 0.2	8.4 ± 0.3	10.2 ± 0.6	8.9 ± 0.4	10.8 ± 0.4	10.8 ± 0.4	11.7 ± 0.3	0.0004	<0.0001
Calcium, mg	902.6 ± 23.0	754.1 ± 16.6	965.9 ± 24.8	799.4 ± 27.6	963.9 ± 31.6	818.3 ± 19.8	1071.1 ± 49.6	852.4 ± 22.5	<0.0001	<0.0001
Iron, mg	11.5 ± 0.2	11.8 ± 0.1	11.0 ± 0.3	12.2 ± 0.4	11.8 ± 0.5	12.8 ± 0.4	14.4 ± 0.5	14.1 ± 0.3	<0.0001	<0.0001
Magnesium, mg	236.1 ± 3.4	280.9 ± 3.9	231.0 ± 7.3	302.3 ± 9.7	262.4 ± 7.1	335.0 ± 6.8	330.8 ± 8.2	388.7 ± 7.8	<0.0001	<0.0001

RAE, retinol activity equivalents. Data are based on the Canadian Community Health Survey (CCHS) 2015 and are presented as mean ± standard error. Results are unadjusted.

[a]CCHS 2015 respondents were stratified according to whole grain intake: those in the "no whole grain intake" (No-WG) group reported consuming no whole grain foods on a single 24-hour dietary recall. The remaining participants that consumed whole grains were divided according to age-specific tertiles into low- middle- and high-whole grain foods intake (low-WG, mid-WG, high-WG).

[b]Statistical significance was determined based on linear trends across whole grain intake groups for children and adults (separately) and a $p<0.002$, after applying a Bonferroni correction for multiple comparisons (0.05/22 nutrients = 0.00227), was considered statistically significant.
