# Supplementary material for: Association between whole grain food intake in Canada and nutrient intake, food group intake and diet quality: Findings from the 2015 Canadian Community Health Survey
Source: PLoS One. 2021 Jul 1;16(7):e0253052. doi: 10.1371/journal.pone.0253052 (PMC8248641; doi:10.1371/journal.pone.0253052)
Supplement: S5 Table — Data are based on the Canadian Community Health Survey (CCHS) 2015 and are presented as mean ± standard error in arbitrary units. Results are unadjusted. (PDF) [file pone.0253052.s005.pdf]

S5 Table. Unadjusted Mean Nutrient Rich Food Index 9.3 for Canadian Children and Adults Stratified by Whole Grain Food Intake.

	**No Whole Grain Food Intake (No-WG)[a]**		**Low Whole Grain Food Intake (Low-WG)[a]**		**Mid-Whole Grain Food Intake (Mid-WG)[a]**		**High-Whole Grain Food Intake (High-WG)[a]**		**p value for linear trend[b]**	
	Children n=3,305	Adults n=7,578	Children n=1,077	Adults n=2,044	Children n=1,085	Adults n=2,156	Children n=1,101	Adults n=2,141	Children	Adults
NRF 9.3 score, arbitrary units[c]	211 ± 4	221 ± 6	257 ± 7	285 ± 7	261 ± 6	290 ± 10	282 ± 6	314 ± 7	<0.0001	<0.0001

Data are based on the Canadian Community Health Survey (CCHS) 2015 and are presented as mean ± standard error in arbitrary units. Results are unadjusted.

[a]CCHS 2015 respondents were stratified according to whole grain intake: those in the "no whole grain intake" (No-WG) group reported consuming no whole grain foods on a single 24-hour dietary recall. The remaining participants that consumed whole grains were divided according to age-specific tertiles into low- middle- and high-whole grain foods intake (low-WG, mid-WG, high-WG).

[b]Statistical significance was determined based on linear trends across whole grain intake groups for children and adults (separately) and a $p<0.05$ was considered statistically significant.

[c]The Nutrient Rich Food Index (NRF) 9.3 is a measure of nutrient density adapted to the total diet that calculates the sum of the contribution to daily recommendations for nutrients to encourage (fiber, protein, vitamin D, vitamin C, iron, calcium, potassium, vitamin A, and magnesium) minus the sum of the contribution to daily recommendations for nutrients to limit (total sugar, sodium, and saturated fat) per 2000 kcals.
