# Supplementary material for: Association between whole grain food intake in Canada and nutrient intake, food group intake and diet quality: Findings from the 2015 Canadian Community Health Survey
Source: PLoS One. 2021 Jul 1;16(7):e0253052. doi: 10.1371/journal.pone.0253052 (PMC8248641; doi:10.1371/journal.pone.0253052)
Supplement: S6 Table — Data are based on the Canadian Community Health Survey (CCHS) 2015 and are presented as the pairwise unadjusted difference in nutrient rich food index 9.3 scores (arbitrary units) and p-values based on the Tukey-Kramer post-hoc test from a multiple linear regression model that included total energy intake (categorical), age (continuous), gender (categorical), overweight/obesity status (yes/no), low-income (categorical), and supplement use (categorical) as covariates. (PDF) [file pone.0253052.s006.pdf]

S6 Table. Pairwise Differences in the Nutrient-Rich Food Index (NRF) 9.3 for Canadian Children and Adults Stratified by Whole Grain Food Intake.

Pairwise Comparisons Between WG Food Intake Groups[a]	**Children 1to 18 Years**		**Adults 19 Years and Older**	
	**Pairwise Difference in mean NRF 9.3 score (arbitrary units)**	**P-Value**	**Pairwise Difference in NRF 9.3 score (arbitrary units)**	**P-Value**
Low-WG vs No-WG	29.7	0.002	53.5	0.0001
Mid-WG vs No-WG	42.5	<.0001	65.4	<.0001
High-WG vs No-WG	73.8	<.0001	90.7	<.0001
Mid-WG vs Low-WG	12.8	0.5	11.9	0.3
High-WG vs Low-WG	44.0	<.0001	37.2	0.07
High-WG vs Mid-WG	31.2	0.0002	25.3	0.4

NRF 9.3, Nutrient-Rich Food Index 9.3. Data are based on the Canadian Community Health Survey (CCHS) 2015 and are presented as the pairwise unadjusted difference in nutrient rich food index 9.3 scores (arbitrary units) and p-values based on the Tukey-Kramer post-hoc test from a multiple linear regression model that included total energy intake (categorical), age (continuous), gender (categorical), overweight/obesity status (yes/no), low-income (categorical), and supplement use (categorical) as covariates.

[a]CCHS 2015 respondents were stratified according to whole grain intake: those in the “no whole grain intake” (No-WG) group reported consuming no whole grain foods on a single 24-hour dietary recall. The remaining participants that consumed whole grains were divided according to age- and sex-specific tertiles into low- middle- and high-whole grain foods intake (low-WG, mid-WG, high-WG).
