# Supplementary material for: Association between whole grain food intake in Canada and nutrient intake, food group intake and diet quality: Findings from the 2015 Canadian Community Health Survey
Source: PLoS One. 2021 Jul 1;16(7):e0253052. doi: 10.1371/journal.pone.0253052 (PMC8248641; doi:10.1371/journal.pone.0253052)
Supplement: S7 Table — Data are based on the Canadian Community Health Survey (CCHS) 2015 and are presented as mean ± standard error. The results are unadjusted. The units for all results are in grams. (PDF) [file pone.0253052.s007.pdf]

S7 Table. Unadjusted Canadian Food Guide Food Group Intake for Children and Adults Stratified by Whole Grain Food Intake.

Food Groups by Canada Food Guide Tiers[c]	**No Whole Grain Food Intake (No-WG)[a]**		**Low Whole Grain Food Intake (Low-WG)[a]**		**Mid-Whole Grain Food Intake (Mid-WG)[a]**		**High-Whole Grain Food Intake (High-WG)[a]**		**p value for linear trend[b]**	
	Children n=3,305	Adults n=7,578	Children n=1,077	Adults n=2,044	Children n=1,085	Adults n=2,156	Children n=1,101	Adults n=2,141	Children	Adults
Whole Grains Tier 1-3, g/d	0 ± 0	0 ± 0	18 ± 1	27 ± 1	52 ± 1	69 ± 1	156 ± 6	216 ± 12	<0.0001	<0.0001
Whole Grains Tier 4, g/d	0 ± 0	0 ± 0	2 ± 0	2 ± 0	3 ± 0	3 ± 0	7 ± 1	4 ± 1	<0.0001	<0.0001
Fruit CFG tiers 1-3, g/d	274 ± 9	196 ± 5	274 ± 13	217 ± 9	308 ± 24	235 ± 10	311 ± 17	243 ± 11	0.01	<0.0001
Fruit CFG tier 4, g/d	0 ± 0	0 ± 0	0 ± 0	0 ± 0	0 ± 0	0 ± 0	0 ± 0	0 ± 0	1.0	0.9
Vegetables CFG tiers 1-3, g/d	150 ± 4	219 ± 8	131 ± 11	220 ± 8	135 ± 11	228 ± 8	152 ± 6	239 ± 15	0.6	0.3
Vegetables CFG tier 4, g/d	3 ± 0	4 ± 1	2 ± 0	4 ± 1	2 ± 1	5 ± 1	4 ± 2	2 ± 1	0.7	0.3
Refined grains CFG tiers 1-3, g/d	199 ± 5	186 ± 7	153 ± 7	145 ± 5	144 ± 11	127 ± 13	145 ± 7	122 ± 4	<0.0001	<0.0001
Refined grains CFG tier 4, g/d	42 ± 4	23 ± 1	35 ± 4	19 ± 6	32 ± 5	23 ± 2	26 ± 4	19 ± 2	0.003	0.1
Dairy products and alternatives CFG tiers 1-3, g/d	348 ± 18	197 ± 7	427 ± 16	240 ± 9	408 ± 18	237 ± 11	403 ± 27	238 ± 13	0.007	0.0008
Dairy products and alternatives CFG tier 4, g/d	6 ± 1	6 ± 1	5 ± 1	5 ± 1	6 ± 1	4 ± 1	4 ± 1	5 ± 1	0.2	0.07
Meat and poultry CFG tiers 1-3, g/d	64 ± 3	86 ± 4	53 ± 6	81 ± 4	48 ± 5	80 ± 5	63 ± 6	73 ± 4	0.2	0.005
Meat and poultry CFG tier 4, g/d	8 ± 1	13 ± 3	4 ± 2	6 ± 1	6 ± 3	6 ± 2	5 ± 2	7 ± 2	0.1	0.003
Processed meats CFG tiers 1-3, g/d	12 ± 1	12 ± 1	9 ± 2	10 ± 1	12 ± 2	12 ± 1	13 ± 2	14 ± 2	0.8	0.3
Processed meats CFG tier 4, g/d	10 ± 1	11 ± 1	11 ± 1	6 ± 1	11 ± 2	9 ± 3	8 ± 1	5 ± 1	0.3	0.01

Food Groups by Canada Food Guide Tiers[c]	**No Whole Grain Food Intake (No-WG)[a]**		**Low Whole Grain Food Intake (Low-WG)[a]**		**Mid-Whole Grain Food Intake (Mid-WG)[a]**		**High-Whole Grain Food Intake (High-WG)[a]**		**p value for linear trend[b]**	
	Children n=3,305	Adults n=7,578	Children n=1,077	Adults n=2,044	Children n=1,085	Adults n=2,156	Children n=1,101	Adults n=2,141	Children	Adults
Fish and shellfish CFG tiers 1-3, g/d	10 ± 1	15 ± 2	8 ± 2	19 ± 4	8 ± 1	18 ± 3	11 ± 2	17 ± 3	0.8	0.2
Fish and shellfish CFG tier 4, g/d	1 ± 0	4 ± 1	1 ± 0	3 ± 1	0 ± 0	4 ± 2	2 ± 1	2 ± 1	0.5	0.2
Legumes, nuts and seeds CFG tiers 1-3, g/d	13 ± 1	22 ± 1	9 ± 1	31 ± 5	18 ± 3	28 ± 3	18 ± 2	39 ± 3	0.03	<0.0001
Legumes, nuts and seeds CFG tier 4, g/d	1 ± 0	1 ± 0	0 ± 0	3 ± 1	1 ± 0	1 ± 1	1 ± 0	2 ± 1	0.9	0.3
Eggs CFG tiers 1-3, g/d	14 ± 1	22 ± 1	13 ± 2	20 ± 2	14 ± 2	26 ± 2	13 ± 2	23 ± 2	1.0	0.2
Eggs CFG tier 4, g/d	0 ± 0	1 ± 0	0 ± 0	0 ± 0	0 ± 0	1 ± 0	0 ± 0	0 ± 0	0.8	0.3
No CFG - Discretionary foods, g/d	181 ± 10	335 ± 15	106 ± 12	226 ± 14	142 ± 15	244 ± 19	155 ± 11	198 ± 13	0.04	<0.0001
No CFG - Other foods and recipes, g/d	770 ± 21	1468 ± 26	655 ± 24	1535 ± 54	741 ± 45	1594 ± 58	883 ± 44	1601 ± 77	0.09	0.1
No CRF – Foods not classified, g/d	573 ± 12	717 ± 10	507 ± 22	706 ± 19	567 ± 24	737 ± 31	599 ± 21	761 ± 28	0.4	0.05

CFG, Canada Food Guide; WG, whole grains. Data are based on the Canadian Community Health Survey (CCHS) 2015 and are presented as mean ± standard error. The results are unadjusted. The units for all results are in grams.

[a]CCHS 2015 respondents were stratified according to whole grain intake: those in the "no whole grain intake" (No-WG) group reported consuming no whole grain foods on a single 24-hour dietary recall. The remaining participants that consumed whole grains were divided according to age- and sex-specific tertiles into low- middle- and high-whole grain foods intake (low-WG, mid-WG, high-WG).

[b]Statistical significance was determined across whole grain intake groups for children and adults (separately) and a p<0.002, after applying a Bonferroni correction for multiple comparisons (0.05/24 food groups = 0.00208), was considered statistically significant.

[c]The four tiers indicate whether a food item is aligned (tiers 1 & 2), partially aligned (tier 3) or not aligned (tier 4) with the 2007 Canadian Food Guide.
