# Supplementary material for: Association between whole grain food intake in Canada and nutrient intake, food group intake and diet quality: Findings from the 2015 Canadian Community Health Survey
Source: PLoS One. 2021 Jul 1;16(7):e0253052. doi: 10.1371/journal.pone.0253052 (PMC8248641; doi:10.1371/journal.pone.0253052)
Supplement: Attachment — Submitted filename: PLOS ONE Reponse to Reviewers R1.docx [file pone.0253052.s008.docx]

**We appreciate the feedback from the editors and reviewers on our manuscript “Association between whole grain food intake in Canada and nutrient intake, food group intake and diet quality: Findings from the 2015 Canadian Community Health Survey.” We’ve made extensive edits to our manuscript based on the comments we received including updates to our formatting; additional details to our methodology; and revised results. These revisions did not, however, materially alter the messages from our paper or our conclusion. We’ve provided point-by-point responses to the editor’s and reviewers’ comments below and indicated the line number, table or figure where the manuscript was edited.**

When submitting your revision, we need you to address these additional requirements.

**1**. Please ensure that your manuscript meets PLOS ONE's style requirements, including those for file naming. The PLOS ONE style templates can be found at

<https://journals.plos.org/plosone/s/file?id=wjVg/PLOSOne_formatting_sample_main_body.pdf> and

<https://journals.plos.org/plosone/s/file?id=ba62/PLOSOne_formatting_sample_title_authors_affiliations.pdf>

***Authors’ response: The formatting of the manuscript has been updated in accordance with the PLOS ONE style requirements.***

**2**.Thank you for stating the following in the Competing Interests section:

"All authors have read the journal's policy and the authors of have the following competing interests: All authors are employed by General Mills, a food manufacturer." Please confirm that this does not alter your adherence to all PLOS ONE policies on sharing data and materials, by including the following statement: "This does not alter our adherence to PLOS ONE policies on sharing data and materials.” (as detailed online in our guide for authors [http://journals.plos.org/plosone/s/competing-interests](about:blank)).  If there are restrictions on sharing of data and/or materials, please state these. Please note that we cannot proceed with consideration of your article until this information has been declared.

 Please include your updated Competing Interests statement in your cover letter; we will change the online submission form on your behalf.

***Authors’ Response: We have updated the statement in our cover letter to reflect that this disclosure does not alter our adherence to PLOSE ONE policies on sharing data and materials.***

**3**. In statistical methods, please refer to any post-hoc corrections to correct for multiple comparisons during your statistical analyses. If these were not performed please justify the reasons. Please refer to our statistical reporting guidelines for assistance (<https://journals.plos.org/plosone/s/submission-guidelines.#loc-statistical-reporting>).

***Authors’ response: We have updated our “Methods” section (lines 193 to 198) to reflect that we used a Bonferroni correction for multiple comparisons for our results presented in Table 2 on nutrient intakes (intake of 22 nutrients; 0.05/22 = 0.00227) and Table 3 on food group intakes (intake of 24 food group; 0.05/24 = 0.00208). Because we were analyzing children and adult populations separately and were not comparing the two age groups to one another we only corrected for multiple comparison within the same population.***

***Three results in Table 2, and four results in Table 3, that were previously significant were no longer statistically significant. Our results section on Nutrient Intakes and Food Group Intakes was updated to reflect these changes; however, these changes did not materially alter our conclusion.***

4. In your statistical analyses, please state whether you accounted for clustering by locality. For example, did you consider using multilevel models?

***Authors’ response: we did not account for clustering by locality. The primary reason for multi-level/hierarchical modeling is to account for subject-level variability and correlations due to group membership. The 2015 CCHS Nutrition public use microfile data, which are publicly available and accompanied by a*** [***User Guide***](https://www.canada.ca/en/health-canada/services/food-nutrition/food-nutrition-surveillance/health-nutrition-surveys/canadian-community-health-survey-cchs/reference-guide-understanding-using-data-2015.html)***, is designed to be a representative survey of the population of Canadians 1 year of age and over living in private dwellings in the ten provinces of Canada. A complex multi-stage, clustered design was used for participant selection so that survey participants would be representative of the Canadian population across urban, rural, age, gender, and socioeconomic characteristics for each province. Dietary intake and other measured characteristics of each study participant would be independent of each other as only one household member was selected to be the study respondent within this clustered survey design. Since the Canadian Community Health Survey is constructed to be representative, we elected to use a simpler, non-hierarchical approach with survey weights. Any similarities in food intake due to regional culture or cuisine is inherent to any regional or national dietary survey and doesn’t invalidate the assumption of independent samples. Therefore, we have not used a multilevel model to analyze our data. This approach is aligned with other publications using this data (see below). We’ve added additional details to the methods section to reflect the survey method and our assumptions (lines 190-191).***

<https://www.ncbi.nlm.nih.gov/pmc/articles/PMC6770056/>

<https://pubmed.ncbi.nlm.nih.gov/31426505/>

<https://pubmed.ncbi.nlm.nih.gov/30408076/>

<https://pubmed.ncbi.nlm.nih.gov/32644762/>

5. We noted in your submission details that a portion of your manuscript may have been presented or published elsewhere.

"These results have been submitted as an abstract to the 2021 American Heart Association Epidemiology and Lifestyle Meeting. The abstract has not yet been accepted or published."

Please clarify whether this [conference proceeding or publication] was peer-reviewed and formally published. If this work was previously peer-reviewed and published, in the cover letter please provide the reason that this work does not constitute dual publication and should be included in the current manuscript.

***Authors’ response: This is not a conference proceeding or full publication but an abstract that was submitted to a conference for a poster presentation. Only the 200 word abstract will be published, which does not undergo a full peer-reviewed.***

6. We note you have included a table to which you do not refer in the text of your manuscript. Please ensure that you refer to Table 4 in your text; if accepted, production will need this reference to link the reader to the Table.

***Authors’ response: We’ve added a reference to Table 4 at two locations in our text: at line 327 and 336.***

Reviewers' comments:

Reviewer's Responses to Questions

**Comments to the Author**

1. Is the manuscript technically sound, and do the data support the conclusions?

The manuscript must describe a technically sound piece of scientific research with data that supports the conclusions. Experiments must have been conducted rigorously, with appropriate controls, replication, and sample sizes. The conclusions must be drawn appropriately based on the data presented.

Reviewer #1: No

Reviewer #2: Yes

2. Has the statistical analysis been performed appropriately and rigorously?

Reviewer #1: No

Reviewer #2: No

***Authors’ response: We have added additional details to our methods section and updated our analyses based on the reviewers’ comments below.***

3. Have the authors made all data underlying the findings in their manuscript fully available?

The [PLOS Data policy](http://www.plosone.org/static/policies.action#sharing) requires authors to make all data underlying the findings described in their manuscript fully available without restriction, with rare exception (please refer to the Data Availability Statement in the manuscript PDF file). The data should be provided as part of the manuscript or its supporting information, or deposited to a public repository. For example, in addition to summary statistics, the data points behind means, medians and variance measures should be available. If there are restrictions on publicly sharing data—e.g. participant privacy or use of data from a third party—those must be specified.

Reviewer #1: No

***Authors’ response: We are using publicly available data that can be downloaded and accessed through Statistics Canada. We have moved these details to the start of our “Methods” section at lines 70-72 and included a reference.***

Reviewer #2: Yes

4. Is the manuscript presented in an intelligible fashion and written in standard English?

PLOS ONE does not copyedit accepted manuscripts, so the language in submitted articles must be clear, correct, and unambiguous. Any typographical or grammatical errors should be corrected at revision, so please note any specific errors here.

Reviewer #1: Yes

Reviewer #2: Yes

5. Review Comments to the Author

Please use the space provided to explain your answers to the questions above. You may also include additional comments for the author, including concerns about dual publication, research ethics, or publication ethics. (Please upload your review as an attachment if it exceeds 20,000 characters)

Reviewer #1: Thank you for the invitation to review this manuscript. Overall, this is an interesting research topic but may need substantial revision. I have highlighted several major methodological concerns below that require the authors’ attention. There were also minor comments that did not include here as they are od lesser importance and I have prioritised the major ones.

Major comments
1. Introduction – comprehensive and sufficient

2. Methods
a. Please describe how dietary recall was done in children. Were proxies used?

***Authors’ response: Yes, proxies were used to report the dietary intake for children 1 to <6 years. Children 6 to 11 years were assisted in completing the dietary recall by a parent or guardian and children over 12 years of age completed the survey on their own. These details have been added to our “Methods” section at lines 86 to 88.***

b. Why was 1 X 24h recall used instead of both recalls? This is an important point esp. the analyses were predominantly based on micronutrients, that vary significantly from day-to-day? Usual intake (instead of actual) intake may be a better reflection of habitual intake, and more accurate representation of diet quality.

***Authors’ response: Only a subset of study participants (n=7,608) were selected to provide a second dietary recall. In order to maintain the full sample size of the study, we used only the first 24 hr dietary recall which was available for all study participants.***

***Further, a single day of dietary record provides a reliable estimate of mean usual intakes, which aligns with our study objectives.*** [***https://dietassessmentprimer.cancer.gov/profiles/recall/***](https://dietassessmentprimer.cancer.gov/profiles/recall/) ***And because we are investigating the associations between whole grain intake and dietary outcomes, a single record can provide insights into how whole grain foods consumed on that day impact dietary outcomes on that same day (we wouldn’t anticipate any lag time between our exposure of whole grain intake and our outcome of nutrient intakes and dietary quality). This is in contrast to health outcomes where it would be more appropriate to approximate usual dietary intake since usual dietary intake is likely to be the relevant exposure for these types of outcomes and the temporal relationship between usual dietary exposure and health outcomes is much longer. We’ve added additional context to lines 84 to 85 of our “Methods” section and our “Strengths and limitations” section at lines 466 to 468.***

c. What was the difference between CFS and BNS food groups? Why was BNS needed if the CFS provides top WG sources too?

***Authors’ response: We provided further details on the differences between the CFG and BNS food groups in our “Methods” section:***

- ***The CFG food groups categorized individual food codes according to their food group and their nutritional composition (i.e. their tiers). We relied on the CFG food groups to provide insights into how whole grain intake was associated with recommended food groups within the Canadian Food Guide. This information is complementary to the Nutrient Rich Food Index (NRF), used to measure dietary quality. (lines 135 to 139)***
- ***BNS food groups categorize individual food codes according to similarities in their dietary usage and nutritional content and provides the means a) to categorize and summarize the detailed food and recipe information collected in nutrition surveys and b) to facilitate analyses of the composition of the diet (https://www.ars.usda.gov/northeast-area/beltsville-md-bhnrc/beltsville-human-nutrition-research-center/food-surveys-research-group/docs/dmr-food-categories/). Unlike the CFG food groups, we used the BNS food groups to report the top sources of WG in the diet of Canadians since the BNS food groups provide further granularity into the types of foods consumed rather than their broad food group or alignment with the Canada Food Guide. (lines 146 to 152)***

***If the reviewer is familiar with NHANES dietary data, CFG food groups would be somewhat comparable to the Food Patterns Equivalent Database (FPED) variables and the BNS food groups would be comparable to the “What we eat in America” food categories.***

d. Whole grain determination should be clearly described – did they include all foods from Tier 1-4 or only those from major food groups but not discretionary foods (e.g. high fat high sugar desserts made with wholemeal flour)?

***Authors’ response: Yes, our definition of whole grain food included high fat and high sugar desserts made with wholemeal flour, for example. We choose to include “Grains – whole” tiers 1-4 food as our definition of whole grain foods (“Methods”, line 103 to 105) to capture whole grain intake as completely as possible. We provide examples of the types of whole grains foods for each tier at lines 105 to 109 of our “Methods” section and tier 4 whole grain foods included homemade granola, buttered popcorn and whole wheat fruit and nut cake. Total intake of tier 4 whole grain foods was significantly lower across all tertiles of WG intake groups than tier 1-3 foods (Table 3). Further, including tier 4 foods would, if anything, bias our results toward showing less differences in nutrient intakes and dietary quality. These insights have been added to our discussion at lines 475 to 479.***

What happened to the 49% food such as mixed dish that can contribute to whole grain intake that were not categorised in the CFG?

***We adhered to the definition of WG foods as provided in the CFG food groups variable and, therefore, did not consider WG intake from mixed dishes. However, we did perform a search of individual food code descriptions that included “whole grain,” “whole wheat,” or “wholemeal.” These individual food codes, and the number of participants that reported consuming them are now reported in a new S1 Table. This rationale and reference to S1 Table is provided at lines 109 to 113 in our “Methods” section.***

e. Whole grain was not seen in the CFG Table S1. Please check.

***Authors’ response: This has now been added to S2 Table (renumbered from S1).***

f. Diet quality – how was NRF chosen. Diet quality should consider intake of food groups and adherence to dietary guidelines, instead of just selected nutrients. Can the authors cite studies that NRF has been validated against other diet quality measures such as the healthy eating index etc.?

***Authors’ response: We agree that diet quality scores should ideally include food groups. However, CCHS dietary data does not provided disaggregated quantitative food group intake information that is necessary to calculate a dietary quality score, like the Healthy Eating Index, that includes food groups. The CFG food groups, as previously noted, do not consider intake of food groups from mixed dishes, which may make a substantial contribution to some food groups, particularly vegetables and dairy, thus biasing the HEI (or other similar) score. Therefore, we choose to use an index of dietary quality that relied on nutrient intake only and reflected the balance of nutrients to encourage and nutrients to limit (sugar, sodium, saturated fat) normalized per calorie (in our case, per 2000kcal) similar to the HEI (which is normalized to 1000kcal). NRF has been reported to be strongly correlated with the HEI. Further, we have provided supporting evidence on the association between WG and food group intake in Table 3. We have added additional details on our rationale for selecting the NRF to our “Methods” section at lines 153 to 155 and added content to our “Strengths and Limitations” section regarding our NRF score at lines 473 to 476.***

g. Has the Nutrient-Rich Food Index 9.3 validated for children, or some adults whose intakes were below 2000 kcal/day?

***Authors’ response: The NRF has been validated against the HEI in children and adults across the full range of calorie intakes available in NHANES (the database used to validate the NRF). It has also been used in numerous studies, including in CCHS, to measure diet quality in both children and adults. This information, including additional references, has been added to the “Diet Quality” subsection in the “Methods” section.***

When standardising dietary intake to 2000 kcal, was it a fair assumption that intake of nutrients and whole grain increased or decreased linearly during (upward or downward) energy standardisation?

***We choose 2000kcal arbitrarily. The score can be normalized to any calorie amount as the intent is to measure the quality of the diet rather than the quantity of the diet and is the same approach used by the HEI (which normalizes intake of nutrients and food groups to 1000 kcal). The score is calculated for each individual so we’re not applying any assumptions about how whole grain or nutrient intakes would increase or decrease (linearly or otherwise) in a model. This is similar to reporting the mean percent of energy coming from macronutrients.***

h. Methods should clearly describe how the ‘moderation’ component scores are being deducted (e.g. only proportion that exceeded recommendation)

***Authors’ response: Nutrient intakes positively contribute to the diet when they are neither too low nor too high. Nutrients to encourage can only contribute a maximum of 100% to the NRF 9.3 score to avoid skewing scores based on very high intakes of a single nutrient. However, we did not apply this cap to the nutrients to limit (sodium, saturated fat, and sugar) such that high intake of these nutrients would reduce the diet quality score. These approaches are aligned with dietary recommendations and public health nutrition principles. Additional details have been added to the “Diet Quality” subsection of the “Methods” section.***

3. Analytical methods for children and adults should be considered carefully:
a. Covariates used in analysis – are they all appropriate/relevant for children?

***Authors’ response. Yes, they are all appropriate and relevant for children. We selected our covariates a priori based on their plausible, and well established, association with nutrient intake, food group intake and diet quality. This has been added to lines 174 to 175 of our “Methods” section.***

b. Covariates – was there an overlap in adjusting sex, BMI, and EI (e.g. females or lean individuals = lower EI)?

***Authors’ response: There is likely some correlation between these variables, however, they are unlikely to be 100% correlated to one another, and therefore their separate inclusion in the model is relevant since they may represent a unique confounding variable when assessing the association between WG intake and the outcomes of interest. All of our unadjusted results are available in the supplemental materials and we found very similar patterns between our adjusted and unadjusted results suggesting that our results are not being biased by “overadjustment.”***

Was physical activity level considered in adjusting dietary intake?

***Authors response: No. We’ve added percent of children and adults meeting their respective physical activity recommendations in Table 1 but have not included physical activity as one of our covariates in our models. Our covariates were selected a priori based on their plausible associations with our outcomes of interest (“Methods” section lines 173 to 174) and physical activity is likely more appropriate when examining health outcomes rather than dietary outcomes.***

Was NRF adjusted, if yes, was EI over-adjusted if NRF already considered per 2000 kcal intake?

***Authors’ response: Yes, NRF was adjusted, including for energy intake. Calorie intake could remain a confounding factor even when consider nutrient intake per 2000 kcal. When comparing the unadjusted to the adjusted NRF results we see similar patterns of results, suggesting that we did not “overadjust” our model. Unadjusted NRF 9.3 results have been added as S5 Table and are referenced in the “Results” section at lines 27 to 271.***

c. Post-hoc analysis could be performed to show participants which WG categories the significant differences came from.

***Authors’ response: because our groups are categorized based on an ordinal variable – i.e. 4 groups going from no whole grain intake to high whole grain intake – we felt it was most appropriate to report the p value based on a test for trend across the 4 groups, providing evidence that there may be a dose-response between our exposure and outcomes. Reporting the results of 6 possible pairwise comparisons for each of our 22 nutrients and 24 food groups would be cumbersome and add little to the interpretation of the results or in understanding their relevance. Details have been added to the “Methods” section at line 192 and the “Strengths and Limitations” section at line 446 to 449.***

4. Results
a. Overall, findings (demographics, nutrient intake, diet quality) should be analysed and reported separately for children and adults, and some reasons are (but not limited to) listed above.

***Authors’ response: We updated Table 1 (demographic characteristics) to present demographic characteristics for children and adults separately. Table 2 (nutrient intakes), Table 3 (food groups) are already presented separately for children and adults. We’ve updated Figure 1 report the NRF 9.3 separately for children and adults. Table 4 and Table 5 (newly added) now report top food sources of whole grains separately for children (table 4) and adults (table 5).***

Also, if combined, young children may be categorised into the low whole grain categories due to their lower total energy intake, hence skewed the results reported in this manuscript.

***Authors’ response: We used age-specific tertiles to classify children and adults into the three categories of whole grain intake (lines 112 to 113 of “Methods” section). We had even distribution of both children and adults across our 3 whole grain intake groups with 16%, 17% and 17% of all children being in the low-WG, mid-WG and high-WG groups, respectively. For adults, we found that 15% of participants were in each of the WG intake groups. Also, in Table 3, the mean intake of Whole Grain intake was lower for children compared to adults at each WG intake level based on the fact that we did use the age-specific tertile cut-offs. We have added additional details to our “Methods” section at lines 116 and 117 and “Results” section at lines 201 to 205.***

b. I suggest to report unadjusted nutrient intake, food group, and diet quality intake but include p values for the adjusted stats.

***Authors’ response: Unadjusted results can be found in the supplemental materials. Adjusting for covariates can at times alter the directionality of relationships between exposures and outcomes; therefore, we felt that reporting both the adjusted and the unadjusted mean values was the most transparent way to communicate our findings.***

c. Energy intake and whole grain should be included in Table 2 for each WG category according to children and adults.

***Authors’ Response: Energy intake has been added to Table 2. Whole grain intake is in Tables 1 and 3.***

d. The authors stated that higher WG was seen with higher EI because of higher overall intake. In this case, could the intake of macronutrients be reported as a percentage of total energy intake? Just a thought.

***Authors’ response: Added to Table 2 and “Nutrient Intakes” subsection of the “Results” section.***

e. Food group results – as above.

5. Discussion
a. Discussion and conclusion need to be revised based on the results after data-re-analysis

***Authors’ response: Discussion has been revised and re-organized. Our conclusion is supported by our data.***

b. Overall lacks depth and the authors could interpret/discuss the findings by considering other food groups too. For examples, could the higher fibre intake found in the high WG group explained by other fibre-rich foods e.g. fruits/vegetables/legumes?

***Authors response: As originally written in our manuscript, at lines 410 to 417:***

These findings highlight the importance of considering the overall nutrient density – that is the balance of nutrients and food groups to encourage and nutrients to limit – rather than only considering nutrients in isolation. Some of the observed differences in nutrient intakes and diet quality may be due to the consumption of whole grain per se but are also likely due to the overall increased intake in other health-promoting food groups including fruits, vegetables, dairy, and nuts and seeds, particularly among foods classified by CCHS as tiers 1-3 (i.e. at least partially recommended by the 2007 CFG). Healthy dietary patterns should be built from nutrient dense foods that include key food groups such as whole grain, dairy, fruit, vegetables, and nuts and seeds.

***We’ve added some additional context at lines 382 to 390 based on the reviewer’s comment:***

*Other food groups may also be contributing to fiber intake as we saw a positive trend for higher fruit and legume, nut and seed intake in adults (but not children) across the four groups of WG intake. However, legume, nut and seed intake was overall much lower than whole grain intake: adults in the high-WG group reported consuming 39g/day of legume, nuts and seeds compared to 216g/d of whole grains. Also, the difference in fruit intake between the no-WG group and high-WG group was 35g compared to a difference in whole grain intake between the two groups of 216g/d. Therefore, while multiple food groups, like fruits, vegetables, legumes, nuts and seeds contribute to fiber intake, in this study, we saw large differences in fiber intake by WG consumption status.*

c. Several epidemiological studies have examined the relationships between WG and nutrients intake (e.g. NHANES, UK National Diet and Nutrition Survey), but these studies were not mentioned in the discussion of findings

***Authors’ response: The following references have been added to an expanded section at the beginning of our “Discussion” section:***

***O'Neil CE, Nicklas TA, Zanovec M, Cho S. Whole-grain consumption is associated with diet quality and nutrient intake in adults: the National Health and Nutrition Examination Survey, 1999-2004. J Am Diet Assoc. 2010;110(10):1461-8. Was cited in our introduction but we’ve added it to our discussion.***

***Hosseini SH, Jones JM, Vatanparast H. Association between Grain Intake, Nutrient Intake, and Diet Quality of Canadians: Evidence from the Canadian Community Health Survey-Nutrition 2015. Nutrients. 2019;11(8). Cited in our discussion***

***We added : Mann, K.D., Pearce, M.S., McKevith, B., Thielecke, F. and Seal, C.J., 2015. Whole grain intake and its association with intakes of other foods, nutrients and markers of health in the National Diet and Nutrition Survey rolling programme 2008–11. British Journal of Nutrition, 113(10), pp.1595-1602.***

d. I suggest the authors to consider separating discussion from results. For example, the authors reported and discussed nutrient intake, which involves food group and diet quality factors that were not reported until much later in the manuscript.

***Authors’ response: We have re-organized our manuscript to have separate “Results” and “Discussion” sections.***

Reviewer #2: The article presents the findings of the Canadian national nutrition survey examining nutrient intake and diet quality of participants with differing wholegrain intake. A less stringent definition of wholegrains were used than provided in the Canadian Dietary Guidelines. It would be interesting to compare how this has impacted the findings and strength of the conclusions.

***Authors’ response: In table 3, we reported that total intake of tier 4 whole grain foods was much lower across all tertiles of WG intake groups than tier 1-3 foods. Further, including tier 4 foods would, if anything, bias our results toward showing less differences in nutrient intakes and dietary quality. These insights have been added to our “Discussion” section at line 475 to 479.***

In addition I have the following comments:
• Were replicate weights and proc surveyreg /proc surveyfreq used for the analysis?

*Authors’ response:* ***Yes*** ***– there are 500 weights in CCHS that were used and we did consider the sampling methods in our analysis using surveyreq in our analysis. This approach is mentioned in our “Methods” section at lines 186 to 188.***

• Misreporting is not described: how many participants misreported energy in the survey? How do the results differ after accounting for misreporting?

***Authors’ response:***

***Authors’ response: We have now included information on the number of participants that potentially under-reported or over-reported calorie intake in our “Methods” section (lines 88 to 90) using cutoffs of under 200 kcal and over 8000 kcal, as has been previously used (***[***https://www.mdpi.com/2072-6643/11/5/1009/htm***](https://www.mdpi.com/2072-6643/11/5/1009/htm)***). Overall, we found very few participants with suspected misreporting of energy and excluding this small number of participants would be unlikely to materially impact our findings. Because we are relying on a single 24 hr recall, as is appropriate for measuring the mean dietary intake of a population, a greater amount of variability around the mean can be expected compared to other dietary assessment methods that measure longer-term usual intake.***

6. PLOS authors have the option to publish the peer review history of their article ([what does this mean?](https://journals.plos.org/plosone/s/editorial-and-peer-review-process#loc-peer-review-history)). If published, this will include your full peer review and any attached files.


If you choose “no”, your identity will remain anonymous but your review may still be made public.


**Do you want your identity to be public for this peer review?** For information about this choice, including consent withdrawal, please see our [Privacy Policy](https://www.plos.org/privacy-policy).

Reviewer #1: No

Reviewer #2: No

[NOTE: If reviewer comments were submitted as an attachment file, they will be attached to this email and accessible via the submission site. Please log into your account, locate the manuscript record, and check for the action link "View Attachments". If this link does not appear, there are no attachment files.]

While revising your submission, please upload your figure files to the Preflight Analysis and Conversion Engine (PACE) digital diagnostic tool, <https://pacev2.apexcovantage.com/>. PACE helps ensure that figures meet PLOS requirements. To use PACE, you must first register as a user. Registration is free. Then, login and navigate to the UPLOAD tab, where you will find detailed instructions on how to use the tool. If you encounter any issues or have any questions when using PACE, please email PLOS at [figures@plos.org](mailto:figures@plos.org). Please note that Supporting Information files do not need this step.
