# Supplementary material for: Association between whole grain food intake in Canada and nutrient intake, food group intake and diet quality: Findings from the 2015 Canadian Community Health Survey
Source: PLoS One. 2021 Jul 1;16(7):e0253052. doi: 10.1371/journal.pone.0253052 (PMC8248641; doi:10.1371/journal.pone.0253052)
Supplement: Attachment — Submitted filename: PLOS ONE Reponse to Reviewers R2.docx [file pone.0253052.s009.docx]

**Comments to the Author**

1. If the authors have adequately addressed your comments raised in a previous round of review and you feel that this manuscript is now acceptable for publication, you may indicate that here to bypass the “Comments to the Author” section, enter your conflict of interest statement in the “Confidential to Editor” section, and submit your "Accept" recommendation.

Reviewer #1: (No Response)

Reviewer #2: All comments have been addressed

2. Is the manuscript technically sound, and do the data support the conclusions?

The manuscript must describe a technically sound piece of scientific research with data that supports the conclusions. Experiments must have been conducted rigorously, with appropriate controls, replication, and sample sizes. The conclusions must be drawn appropriately based on the data presented.

Reviewer #1: Yes

Reviewer #2: Partly

3. Has the statistical analysis been performed appropriately and rigorously?

Reviewer #1: Yes

Reviewer #2: Yes

4. Have the authors made all data underlying the findings in their manuscript fully available?

The [PLOS Data policy](https://nam02.safelinks.protection.outlook.com/?url=http%3A%2F%2Fwww.plosone.org%2Fstatic%2Fpolicies.action%23sharing&data=04%7C01%7C%7Cc774a998ce6746c75cf008d90ae71196%7C0c33cce8883c4ba5b61534a6e2b8ff38%7C0%7C0%7C637552809816102322%7CUnknown%7CTWFpbGZsb3d8eyJWIjoiMC4wLjAwMDAiLCJQIjoiV2luMzIiLCJBTiI6Ik1haWwiLCJXVCI6Mn0%3D%7C1000&sdata=ZonWzyv5QGuHhfg%2BB8A0KztPKYbqen32ZOzZvpc420w%3D&reserved=0) requires authors to make all data underlying the findings described in their manuscript fully available without restriction, with rare exception (please refer to the Data Availability Statement in the manuscript PDF file). The data should be provided as part of the manuscript or its supporting information, or deposited to a public repository. For example, in addition to summary statistics, the data points behind means, medians and variance measures should be available. If there are restrictions on publicly sharing data—e.g. participant privacy or use of data from a third party—those must be specified.

Reviewer #1: Yes

Reviewer #2: Yes

5. Is the manuscript presented in an intelligible fashion and written in standard English?

PLOS ONE does not copyedit accepted manuscripts, so the language in submitted articles must be clear, correct, and unambiguous. Any typographical or grammatical errors should be corrected at revision, so please note any specific errors here.

Reviewer #1: Yes

Reviewer #2: Yes

6. Review Comments to the Author

Please use the space provided to explain your answers to the questions above. You may also include additional comments for the author, including concerns about dual publication, research ethics, or publication ethics. (Please upload your review as an attachment if it exceeds 20,000 characters)

Reviewer #1: The authors have addressed most of my comments (thank you), and I have a few remaining or new comments for the authors' consideration:

1. In the Methods Diet Quality section, the authors state that total sugar was used instead of added sugar (line 165). Does this affect the validity of this score? Total sugar includes sugars from fruits and dairy and it does not make sense to deduct points for the consumption of sugar from these sources compared to added sugar?

**Authors’ response: The publicly available CCHS data did not provide information on added sugar intake so we used total sugar. The nutrient-rich food index has been validated using total sugar in reference 23: Fulgoni, V.L., 3rd, D.R. Keast, and A. Drewnowski, Development and validation of the nutrient-rich foods index: a tool to measure nutritional quality of foods. J Nutr, 2009. 139(8): p. 1549-54.**

2. Regarding response to question 3b, have you checked for co-linearity between sex, BMI and EI?

**Authors’ response: We checked the co-linearity between sex, BMI, and EI, along with our other covariates and found no evidence of co-linearity. VIF values are all very close to 1.0 and tolerance values are all greater than 0.9. Below is the correlation matrix as well for independent variables in total population (1+ years).**

|  | **WG_Cat3** | **DHH_SEX** | **BMI_Cov** | **Income** | **Supplement** | **CalQuart** |
| --- | --- | --- | --- | --- | --- | --- |
| **WG_Cat3** | 1.00 | -0.03 | 0.00 | -0.02 | 0.11 | 0.06 |
| **DHH_SEX** | -0.03 | 1.00 | -0.01 | 0.04 | 0.14 | -0.30 |
| **BMI_Cov** | 0.00 | -0.01 | 1.00 | 0.02 | -0.01 | -0.09 |
| **LowIncome** | -0.02 | 0.04 | 0.02 | 1.00 | -0.07 | -0.06 |
| **Supplement** | 0.11 | 0.14 | -0.01 | -0.07 | 1.00 | -0.04 |
| **CalQuart** | 0.06 | -0.30 | -0.09 | -0.06 | -0.04 | 1.00 |

**We noted at lines 188-189 that we found no evidence of collinearity between our covariates.**

3. I agree that post-hoc comparisons may be too much for nutrients intake, but I suggest the author to do this for the NRF scores. This relates to Point 6 below.

**Authors’ Response: We have done a post-hoc pairwise comparison for the NRF scores. For adults and children, all of the WG intake groups (low, mid, and high) had significantly higher diet quality compared to the no-WG group. For adults, there were no significant differences in diet quality between the low-, mid- and high-WG intake groups; however, for children, the high-WG intake group had significantly higher diet quality compared to the low- and mid-WG intake groups. There was no significant difference between the low-WG and mid-WG intake groups for children.**

**These findings have been added to our “Results” section at lines 282 to 290. We added a supplemental table, S6 Table, that includes the p-values for the post-hoc pairwise comparisons.**

4. Table 2 - Title says 'adjusted' but EI is 'Unadjusted'

Authors’ Response: We moved the unadjusted energy intake results to Table 1 and moved mention of the findings in the “Results” section to lines 213-216.

5. Table 3 (applies to supplement) - can you add a comma before the unit, and should it be g/day?

**Authors’ Response: Done**

6. Discussion line 376-377 - the statement suggests a dose-response relationship, but the diet quality scores do not appear to be different between low- and mid- intake groups. Please add post-hoc comparisons to support this statement if this was indeed the case.

**Authors’ Response: We have added results for a pairwise Post-hoc comparison for the NRF 9.3 results to our “Results” section at lines 282 to 290 and to S6 Table. We removed from the discussion at line 477 the statement that there was a dose-response relationship between whole grain intake and diet quality.**

7. Discussion line 406 - Please clarify what 'complementary' means in term of the prevalence of obesity in this study and Hosseini et al.

**Authors’ Response: We were referring to our results on the association between whole grain and nutrient intake being complementary. To make this more clear, we edited line 431 and 435.**

8. Discussion line 421 - You cited Ref 37 Hosseini but I cannot see diet quality being reported in the cited study?
**Authors’ Response: We updated our reference to the following:** [**https://pubmed.ncbi.nlm.nih.gov/31426505/**](https://pubmed.ncbi.nlm.nih.gov/31426505/) **“Association between Grain Intake, Nutrition Intake, and Diet Quality of Canadians: Evidence from the Canadian Community Health Survey-Nutrition 2015” at lines 429 and 437.**

9. Discussion Line 438 - A previous study also analysed the sources of whole grains in children and adults, see Galea et al, Public Health Nutrition 2017;20(12)"2166-2172.

**Authors’ Response: We have added this citation at line 469, along with two other references from US and France which have also shown that bread and ready-to-eat cereal are the top sources of whole grains. We modified our sentence at line 464 to clarify that these are novel results on the top sources of whole grains specifically for the Canadian data.**

10. Line 458 - in this study there was no significant difference in overweight and obesity prevalence between groups (Table 1) so I think this statement could be removed.

**Authors’ Response: We have removed lines 491 to 495.**

Reviewer #2: 1.Many dietary guidelines recommend that wholegrains compose 50% of grain foods consumed. Can the authors please provide the proportion of grain foods that are wholegrain?

**Authors’ Response: Yes, based on our unadjusted food group intakes, we calculated the mean of the ratio whole grain food intake (tiers 1-3 + tier 4) to total grain intake (defined as refined grains tier 1-3 + refined grains tier 4 + whole grains tier 1-3 + whole grains tier 4). For children, whole grains were 0%, 16%, 33% and 54% of total grain intake for the no-, low-, mid-, and high-WG intake groups, respectively. For adults, whole grains were 0%, 26%, 45% and 65% of total grain intake for the no-, low-, mid-, and high-WG intake groups, respectively.**

**These results have been added to Table 1 and to lines 209 to 213 in the “Results.” The “Methods” section was updated at lines 114 to 118 and we added a paragraph with these findings to our “Discussion” at lines 400 to 405.**

2.The dataset is not disaggregated. How were the amounts of wholegrains in mixed dishes assessed?

**Authors’ Response: The section of Methods starting at line 96 subtitled “Whole Grain Intake” explains how we defined whole grains. In particular, lines 109-113 state “We only considered whole grain intake from foods classified as whole grain according to the CFG food groups, and not whole grain from mixed dishes, which were not classified into CFG food groups. S1 Table includes a list of individual food codes from mixed dishes that included the terms “whole grain,” “whole wheat,” or “wholemeal” in their description.”**

**In the discussion, we have added the following to our limitation section at line 510: “The CCHS did not disaggregate whole grain content from mixed dishes and we were unable to consider whole grain content from those dishes; however, there were few food codes from mixed dishes that included whole grains (or a similar term) in their description and only a small number of participants consumed these mixed dishes suggesting that our definition captured nearly all whole grain intake.”**

3.Please conduct a sensitivity analysis to determine the effect of energy misreporting on the analysis and report the proportion of plausible reporters in Table 1. Please use a method to determine misreporting that has been validated for 1 day of dietary data and cite the paper that validates the method not just previous research that has used the method. Two validated methods include the Goldberg equation or energy prediction equations reported by Huang, 2005, Obesity Res.

**Authors’ Response: We have chosen to provide an estimate of unrealistic reporting of calorie intake based on reports of <200 and >8000 kcal/day which can be found in our method section at lines 88 to 90. The reviewer has not provided a rationale for requiring this information in a different format. We feel that our approach for characterizing potential misreporting is appropriate based on the following:**

1) **There is no ideal approach to identifying under-reporting – a publication noted that there was only a 50% sensitivity for the Goldberg equation to correctly identify underreporting from 24 hr recalls (https://www.ncbi.nlm.nih.gov/pmc/articles/PMC3319469/).**
2) **Because someone reports less energy intake on a single 24 hr recall than what they typically consume does not mean that they are mis-reporting their dietary intake and we have no way to make this distinction with the data available. We are using the 24 hr recalls to estimate mean population intakes and are not attempting to estimate usual dietary intake in individuals.**
3) **We are not associating dietary intake with health outcomes such as obesity, changes in body weight or with other health outcomes where removing under- or over-reported dietary intake would be more appropriate. Nor are we looking at trends in intake over time, where changes in misreporting could impact trends.**

**We acknowledge that under-reporting is a source of bias in observational nutritional research and have added the following to our discussion section at lines 482 to 490:**

**“While misreporting of energy intake has been well-documented in self-reported dietary intakes, we found that very few participants in our study reported extreme calorie intakes below 200 kcal or above 8000 kcal. Tugault-Lafleur et al, using a different approach to classify mis-reporters, estimated in the CCHS 2015 that under-report of energy intake was 30% and overreporting of energy intake was 9%. While there is no one “correct” way to identify mis-reporting in self-reported dietary intake studies, the potential for mis-reporting should be considered when interpreting these results; however, this study is not attempting to estimate usual energy intake nor assess the association between dietary intake and health outcomes, where usual energy intake and energy misreporting would be more relevant.”**

4.It would be of greater value to the literature to know which subgroups of the population have a diet composition composed of less wholegrain relative to requirements. This will better reflect which groups should increase intake to meet recommended amounts of wholegrains. I suggest categorizing participants as grams per 1000 kcal as suggested: <https://dietassessmentprimer.cancer.gov/learn/adjustment.html>.
**Authors’ response: We agree that this would be interesting and relevant information. We have not included this information because**

**1) There are no quantitative whole grain recommendations in Canada against which to compare intakes.**

**2) CCHS data does not provide disaggregated whole grain information; therefore, we’d need to estimate the whole grain content of each individual food code.**

**Therefore, it is currently out of scope for this research paper to calculate the percent of the Canadian population meeting whole grain intake recommendations using CCHS data but agree that it would be a great line of future research and we have noted this in our discussion at lines 519 to 525.**

5. It is a limitation that grams rather than serves are used to assess wholegrain intake. Is there a reason why serves couldn't be assessed?

**Authors’ Response: The CCHS 2015 does provide a pre-defined variable for the number of servings of CFG whole grain foods. We have now reported the mean number of servings of whole grain tier 1-4 foods in Table 1 and at lines 210 to 213 in the results: the mean number of servings ranged from 0.6 servings/day in the low-WG group to 3.4 servings/day in the high-WG group for children and from 0.9 servings/day in the low-WG group to 3.7 servings/day in the high-WG group for adults. We also mention these findings in our “Discussion” at lines 400 to 405.**

6. It should be argued that participants that consumed more wholegrains also consumed more of other nutrient-dense foods and therefore increasing wholegrains is not necessarily an independent component to contribute to better diet quality based on the present analysis. The conclusion should be altered accordingly

**Authors’ Response: We did note this in our discussion at several points:**

1) **At lines 440 to 443 “Some of the observed differences in nutrient intakes and diet quality may be due to the consumption of whole grain *per se* but are also likely due to the overall increased intake in other health-promoting food groups including fruits, vegetables, dairy, and nuts and seeds, particularly among foods classified by CCHS as tiers 1-3 (i.e. at least partially recommended by the 2007 CFG).”**
2) **at lines 478 to 479 we wrote “due to the cross-sectional and observational study design, causation cannot be assigned”**
3) **and at lines 498 to 499 we wrote “While we adjusted our outcomes for some of these important demographic characteristics, there remains the possibility of residual confounding, inherent to all observational research.”**

**In our conclusion at lines 530 to 533, we appropriate noted that our findings were based on associations. We wrote “We found that higher whole grain intake was associated [emphasis added] with higher intake of key nutrients, particularly fiber, potassium and iron, and associated with higher diet quality. Intake of whole grain was associated with higher intake of other recommended food groups including fruits, dairy, and legumes, nuts and seeds.”**

**We modified lines 533 to 534 to make it clearer that our results support already existing recommendations to consume more whole grains and removed any implications that whole grains could be causally related to improved diet quality.**

7. PLOS authors have the option to publish the peer review history of their article ([what does this mean?](https://nam02.safelinks.protection.outlook.com/?url=https%3A%2F%2Fjournals.plos.org%2Fplosone%2Fs%2Feditorial-and-peer-review-process%23loc-peer-review-history&data=04%7C01%7C%7Cc774a998ce6746c75cf008d90ae71196%7C0c33cce8883c4ba5b61534a6e2b8ff38%7C0%7C0%7C637552809816112318%7CUnknown%7CTWFpbGZsb3d8eyJWIjoiMC4wLjAwMDAiLCJQIjoiV2luMzIiLCJBTiI6Ik1haWwiLCJXVCI6Mn0%3D%7C1000&sdata=GF4FXZkj1IsaIVhK6Pp8Y29t%2Fpi8yL95LIDn2wjwVys%3D&reserved=0)). If published, this will include your full peer review and any attached files.


If you choose “no”, your identity will remain anonymous but your review may still be made public.


**Do you want your identity to be public for this peer review?** For information about this choice, including consent withdrawal, please see our [Privacy Policy](https://nam02.safelinks.protection.outlook.com/?url=https%3A%2F%2Fwww.plos.org%2Fprivacy-policy&data=04%7C01%7C%7Cc774a998ce6746c75cf008d90ae71196%7C0c33cce8883c4ba5b61534a6e2b8ff38%7C0%7C0%7C637552809816112318%7CUnknown%7CTWFpbGZsb3d8eyJWIjoiMC4wLjAwMDAiLCJQIjoiV2luMzIiLCJBTiI6Ik1haWwiLCJXVCI6Mn0%3D%7C1000&sdata=9NWUt1ajLcNmpZH%2FN4w%2BNWJ4ml%2FAntgML1tndbg9MfA%3D&reserved=0).

Reviewer #1: No

Reviewer #2: No
